# Assisted Reproductive Technologies (ARTs) in Domestic Mammals

**DOI:** 10.3390/vetsci10040287

**Published:** 2023-04-11

**Authors:** Salvador Ruiz, Juan Carlos Gardón, Jordi Miró

**Affiliations:** 1 Department of Physiology, Faculty of Veterinary Medicine, Campus Mare Nostrum, University of Murcia, 30100 Murcia, Spain; 2 Institute for Biomedical Research of Murcia, IMIB-Arrixaca, 30120 Murcia, Spain; 3 Department of Animal Medicine and Surgery, Faculty of Veterinary and Experimental Sciences, Catholic University of Valencia-San Vicente Mártir, Guillem de Castro 106, 46003 Valencia, Spain; 4 Equine Reproduction Service, Department of Animal Medicine and Surgery, Faculty of Veterinary Sciences, Autonomous University of Barcelona, 08193 Cerdanyola del Vallès, Spain

As guest editors, we are pleased to present this Special Issue entitled “Assisted Reproductive Technologies (ARTs) in Domestic Mammals”, comprising 10 articles of relevant interest in the field of animal reproduction. Eight research articles [1,2,3,4,5,6,7,8], one feature paper [7], and two review papers [9,10] were included.

In these studies, different experiments were carried out in the area of biotechnology for animal reproduction using various animal models, including sheep [1,10], sows [2], mares [7], bulls [3,4,9], donkeys [5], boars [6], and stallions [8].

Researchers from Pakistan [1], China [1,9,10], Spain [2,3,5,6,8], France [3], Poland [4], Portugal [4], Saudi Arabia [4], Italy [5], Brazil [7], USA [7], and South Africa [9] conducted these trials, and they present their latest findings in this Special Issue. Notably, all 10 articles involve various research centers, and 6 studies are the result of collaboration among different countries [1,3,4,5,7,9], which demonstrates the high degree of international collaboration in this field.

In summary, this Special Issue provides new insights into the application of biotechnology for animal reproduction by presenting the most recent advances and latest research findings from around the world.
